# Using the Reach, Effectiveness, Adoption, Implementation, and Maintenance framework to evaluate a state-wide safe infant sleep education program for continuous improvement

**DOI:** 10.3389/fpubh.2025.1540451

**Published:** 2025-08-13

**Authors:** Carolyn R. Ahlers-Schmidt, Christy Schunn, Ashley M. Hervey, Maria Torres, Stephanie Kuhlmann, Zachary Kuhlmann

**Affiliations:** ^1^Center for Research for Infant Birth and Survival, Wichita, KS, United States; ^2^Department of Pediatrics, University of Kansas School of Medicine-Wichita, Wichita, KS, United States; ^3^Kansas Infant Death and SIDS (KIDS) Network, Wichita, KS, United States; ^4^Department of Obstetrics and Gynecology, University of Kansas School of Medicine-Wichita, Wichita, KS, United States

**Keywords:** RE-AIM framework, implementation science, safe sleep education, sudden unexpected infant death (SUID), sleep-related death

## Abstract

**Introduction:**

The Kansas Infant Death and SIDS (KIDS) Network facilitates a two-day conference certifying Safe Sleep Instructors to provide standardized trainings based on the American Academy of Pediatrics (AAP) Safe Sleep Recommendations. Within one-year of certification, Safe Sleep Instructors are tasked with (a) disseminating safe sleep education to 10 professionals or parent/caregivers; and (b) hosting one Safe Sleep Community Baby Shower or 10 Safe Sleep Crib Clinics.

**Methods:**

A retrospective study was implemented to assess the impact of the Safe Sleep Instructor certification program using data from participants trained in Fiscal Year 2022. Data was assessed using the RE-AIM framework and aspects of the Kirkpatrick Evaluation Model. Reach and Effectiveness were evaluated at the conference, as were Kirkpatrick Reaction and Learning. Adoption and Implementation, and Kirkpatrick Behavior, were assessed through post-conference activities. Maintenance was measured based on Safe Sleep Instructor recertification the following year.

**Results:**

Results suggested the Safe Sleep Instructor certification was successful in terms of Reach, Effectiveness, Implementation and Maintenance, and Kirkpatrick Evaluation Model. Adoption was less than expected.

**Conclusion:**

Utilizing dissemination and implementation science allowed for identification of strengths and limitations regarding the Safe Sleep Instructor program promoting the AAP Safe Sleep Recommendations. Modification to program requirements and expectations, post-conference support for activities, and increased ability to tailor to community needs may enhance Adoption efforts. In addition, Safe Sleep Instructors must have capacity, resources, expertise, and institutional support. Future studies are needed to assess characteristics that impact Adoption to enhance promotion of the AAP Safe Sleep Recommendations.

## 1 Introduction

Since the 1990s the American Academy of Pediatrics (AAP) has recommended evidence-based interventions (EBI) to reduce sudden unexpected infant death (SUID). Recommendations include strategies such as placing infants in supine position, on a safety-approved sleep surface (e.g., bassinet, crib) in a tobacco-free environment (1, 2). Despite known risk reduction strategies, SUID is the leading cause of mortality between 28 days and 1 year of life, impacting 3,400 infants annually in the United States (3).

Most SUIDs, also termed sleep-related infant deaths, include external risk factors which may have contributed to the death (3). While public awareness campaigns (4) and community-based interventions (5–7) promote knowledge and address barriers, the burden of providing safe sleep interventions to families often falls on physicians and clinicians (e.g., nurses, midwives) (2). Efforts in the clinical setting have had some success, with families who receive advice from healthcare professionals more likely to report following safe sleep practices (8–10). However, many factors impact safe sleep behaviors including knowledge; convenience; perceived quality of infant sleep; perceived infant safety, comfort, and susceptibility; and cultural factors (11–19). Further, professionals do not always provide complete or consistent safe sleep information (8, 9, 20, 21), and some groups distrust advice from healthcare professionals (8, 11, 17, 22, 23). For example, persons with low income (8, 11, 22, 23) or of Black/African American (8, 17) descent may be wary of advice due to historical discrimination or systemic racism (24). Translational science is needed to determine effective strategies to promote SUID risk-reduction strategies and to address barriers to safe sleep practices (25).

In Kansas, the SUID rate is higher than the national average (120 infant deaths per 100,000 live births compared to 92 infant deaths; 2016–2020 data) (3). To promote SUID risk reduction strategies and ensure consistent safe sleep messages across the spectrum of perinatal services (8, 11, 18, 26, 27), an infrastructure to educate professionals and families across the state was needed. The Kansas Infant Death and SIDS (KIDS) Network and the University of Kansas School of Medicine-Wichita (KUSM-W) Center for Research for Infant Birth and Survival (CRIBS) partnered to develop an innovative strategy to scale-up safe sleep education interventions in Kansas. The Safe Sleep Instructor (SSI) certification program was developed to embed local experts across the state to provide education on the AAP safe sleep recommendations and to promote a culture of safe infant sleep (28).

No identified studies utilize an implementation science framework to assess the impact of interventions to promote uptake of the AAP Safe Sleep Recommendations. As such, the purpose of this study was to assess the impact of the SSI certification program in enhancing dissemination of the AAP Safe Sleep Recommendations across a rural state.

## 2 Materials and methods

### 2.1 Overall design

A retrospective study using the RE-AIM (Reach, Effectiveness, Adoption, Implementation, and Maintenance) framework (29, 30) was implemented based on data from the Safe Sleep Instructor cohort certified during Fiscal Year 2022. In addition, portions of the Kirkpatrick Evaluation Model were employed to clarify training effectiveness (31). This project involved secondary analysis of deidentified program data collected by KIDS Network staff for program reporting purposes; as such, the University of Kansas Medical Center Human Subjects Committee did not require ethics approval for the study.

### 2.2 SSI training program

The 2-day in-person conference to certify new SSIs consisted of didactics, hands-on demonstrations, video, small group activities, expert panel discussions and large group discussions provided by SSI Faculty (CRS, CS, AH, MT, SK, ZK) (26, 32). On day one, the curriculum covered data trends regarding sleep-related infant deaths and the AAP Safe Sleep Recommendations (1, 2). SSI trainees learned how to provide standardized education on the AAP Safe Sleep Recommendations to professionals (professional training) and caregivers (parent/caregiver training). The SSI training also included strategies on how to identify and engage community champions to build organizational partnerships to reach learners. SSI trainees were instructed on how to perform a Safe Sleep Crib Demonstration (33). This involved setting up a portable crib and using safe (e.g., wearable blanket) and unsafe (e.g., loose blanket, pillow) items with a doll to actively demonstrate how to create a safe sleep environment. This hands-on activity allowed SSI trainees to build skills and critically think about application of the AAP Safe Sleep Recommendations, as well as learn a demonstration method to employ when educating about infant safe sleep. SSI trainees engaged in small group dialog related to addressing social and cultural barriers to following the AAP Safe Sleep Recommendations.

On day two, SSI trainees learned how to educate persons in the perinatal period through a Safe Sleep Community Baby Shower (CBS) (26). Curriculum included how to identify and collaborate with key partners (e.g., hospitals, clinics, community programs, churches, local breastfeeding and tobacco cessation experts); identify priority populations (e.g., race/ethnicity, zip code); engage priority populations (e.g., partnering with organizations, community or religious leaders, existing events); identify and understand local infant mortality data; develop grant proposals for material supports (e.g., food/beverage, location rental fees, cribs for attendees); manage event logistics; engage vendors (e.g., hospital, insurance providers, oral health providers); collect data; and conduct evaluations. For SSIs from small, rural communities with access to fewer pregnant persons, or those who need to connect with participants virtually, instructions were shared for hosting a smaller event, referred to as a Safe Sleep Crib Clinic (CC) (33). A bereaved family was also invited to share their experience and to issue a call to action to disseminate safe sleep information.

To be certified, the SSI trainee was required to pass a knowledge assessment (score ≥ 90%) and successfully conduct a Crib Demonstration (score ≥ 90%). Those who did not meet the *a priori* level for certification were provided remediation by KIDS Network staff until they were successful. Once certified, SSIs were expected to complete the following within 1 year: (a) disseminate safe sleep education to at least 10 professionals or parent/caregivers; (b) host a Safe Sleep Community Baby Shower or 10 Safe Sleep Crib Clinics; and (c) submit pre/post-test data for these trainings. No compensation is provided to the SSIs by the KIDS Network for these dissemination efforts.

Each year certified SSIs attend a recertification webinar to learn new information (e.g., data, research, updated AAP recommendations) related to SUID and receive revised training materials to maintain their certification.

### 2.3 Data sources

#### 2.3.1 Conference evaluation

Reach and Effectiveness were evaluated at the 2-day SSI conference using data collected by KIDS Network staff. Reach included the number of new SSIs certified and the number of professions represented in the training. No minimum educational or employment qualifications were required to become an SSI, however priority was given to those employed in a maternal/infant health-related field and who indicated supervisor approval to embed SSI activities into their job duties. SSI training participants were recruited across the state of Kansas through email invitations distributed by the KIDS Network, Kansas Department of Health and Environment (KDHE) and other maternal and infant health partners. Promotion and recruitment were also done at the KDHE Home Visiting Conference, Annual Governor’s Conference for the Prevention of Child Abuse and Neglect, Annual Kansas Governor’s Public Health Conference, Kansas Perinatal Community Collaboratives, Safe Kids Kansas Coalitions, local Integrated Referral and Intake System (IRIS) quarterly meetings, Emergency Medical Services for Children (EMSC) Advisory Committee Meetings, Kansas State University Extension Services and Community Health Worker Program. Communities with high rates of infant mortality, based on vital statistics reports (34) were contacted directly (e.g., health department, hospital, home visiting program, Department of Children and Families) to recruit training participants. In addition, participants external to Kansas were invited through national conference presentations (e.g., National Parents as Teachers Conference; Cribs for Kids; Safe Kids Worldwide Childhood Injury Prevention Convention), the American Association of SIDS Prevention Physicians (AASPP) listserv and meetings with the Minnesota Public Health Department, Halo Innovations, and Mississippi SIDS and Infant Safety Alliance.

Effectiveness was measured using a 10-item SSI Training Test administered pre- and post-conference. The test was developed by SSI faculty to measure knowledge related to the AAP Safe Sleep Recommendations. All participants completed the pre-and post-tests at the beginning of the conference and immediately following. The tests included the same 10 multiple choice questions, in modified order, which addressed the definition of SUID and specific AAP recommendations.

Effectiveness was also measured by SSI trainee ability to complete a Crib Demonstration with at least 90% fidelity. SSI Faculty used a 10-item checklist which included nine priority points to address during the demonstration (e.g., on back, why loose blankets are not safe, wearable blanket as alternative to loose blankets) and a list from which the SSI Faculty selected a supplemental questions to ask (e.g., where to get a crib, how to know if it was safe/had been recalled).

To further assess conference impact a post-conference evaluation was administered to all attendees. Reaction and Learning were assessed based on the Kirkpatrick Evaluation Model (31). For Reaction, relevance of the topics presented, whether the training was worthwhile, and satisfaction were measured, all with 5-point rating scales (5-representing positive endorsement). Learning was measured with 5-point Likert-type scales addressing the following: gained insight into safe infant sleep; materials were understandable; information was new; felt prepared to implement; and likelihood of providing safe sleep education.

#### 2.3.2 Post-conference evaluation

Following the SSI conference, certified SSIs recruited participants to attend professional training(s), parent/caregiver training(s), CBS(s) and/or CC(s) in their communities. Adoption included the percentage of certified SSIs who: (1) conducted one or more Professional Trainings; (2) conducted one or more Parent/Caregiver Trainings; (3a) facilitated one or more CBS; (3b) facilitated one or more CC; (4) facilitated post-conference trainings at requested levels. In addition, Kirkpatrick Behavior examined SSIs who conducted no post-conference trainings (31).

Implementation was measured based on knowledge change between pre-and post-tests for Professional Trainings, Parent/Caregiver Trainings, CBSs, and CCs. All pre- and post-tests were developed by SSI Faculty to measure knowledge related to the AAP Safe Sleep Recommendations.

- Professional Training Tests: 10-item matched pre- and post-tests, similar to the SSI Training Tests but with less complex questions.
- Parent/Caregiver Training Tests: 3-item unmatched pre- and post-test assessed knowledge related to (1) infant sleep position, (2) sleep location and (3) items in the sleep environment.
- CBS/CC Tests: a 3-item matched pre- and post-test of knowledge related to (1) infant sleep position, (2) sleep location and (3) items in the sleep environment were collected.

#### 2.3.3 Maintenance

Finally, Maintenance was measured using data collected by KIDS Network staff regarding the proportion of SSIs certified in Fiscal Year 2022 who attended the recertification training and certified for the following year (Fiscal Year 2023).

### 2.4 Data analysis

KIDS Network staff collected, deidentified and entered conference evaluation data into a secure online database. Post-conference data were collected, deidentified, and entered into a secure online database by certified SSIs following each training. Participants were encouraged to complete the pre-and post-tests in their entirety. However, due to the voluntary nature respondents had the right to skip questions. Missing data were removed from analysis. All reported data were collected for SSIs trained in Fiscal Year 2022 (Fall 2021 cohort: October 1, 2021–September 30, 2022; Spring 2022 cohort: June 1, 2022–May 31, 2023).

Descriptive statistics were summarized using frequencies (percentages) and means (standard deviations). The 10-item SSI Training Tests and Professional Training Tests were coded as correct vs. incorrect. Differences were evaluated by Wilcoxon Signed Rank Test. The 3-item Parent/Caregiver Training Tests were coded as safe vs. unsafe and evaluated by the Mann–Whitney U-Test. The 3-item CB and CC Test responses were coded as safe vs. unsafe, and comparisons were made using McNemar for paired dichotomous variables. Alpha=0.05 for all. Statistical analyses were performed using SPSS for Windows, Version 26.0 (IBM Corp).

## 3 Results

### 3.1 SSI conference

#### 3.1.1 RE-AIM: reach

Sixty-four individuals registered for the Fall 2021 (*n* = 28) and Spring 2022 (*n* = 36) trainings, and 49 (77%) attended. Of these, 21 (43%) attended the Fall training and 28 (57%) attended the Spring training. SSI trainees represented nine professions as detailed in Table 1.

**Table 1:** Safe Sleep Instructor (SSI) training evaluation using RE-AIM Framework.

	Evaluation Question	Indicator	*N*	%	Statistical analysis
SSI conference evaluation					
R	Did the # of certified SSIs increase?	New SSIs certified	49	---	
	Were participants from a variety of professions?	Professions			
		Nurse	12	25%	
		Social Worker	10	20%	
		Other^1^	8	16%	
		Early Childhood Professional	7	14%	
		Other Healthcare Professional	4	8%	
		Social Service Worker	4	8%	
		Emergency Medical Services (EMS) Worker	2	4%	
		Home Visitor	2	4%	
		Parent Educator	2	4%	
E	Did SSI knowledge of AAP Safe Sleep Recommendations improve between pre- and post-assessment?	Pre- and post-assessment	Pre M (SD)	Post M (SD)	t-test, *p*-value
		SSI Training Test (10-item)	8.3 (1.2)	9.4 (0.8)	t = 6.11, *p* < 0.001
	Could trainees conduct a Safe Sleep Crib Demonstration?	Demonstration Observation	*N*	%	
		Score ≥ 90% on 10-item checklist by trained observer	49	100	
SSI post-conference evaluation					
		Certified SSIs	49	100%	
A^2^	Did certified SSIs conduct Professional Trainings after the conference?	Proportion of SSIs who conducted at least one Professional Training	24	49%	
	Did certified SSIs conduct Parent/Caregiver Trainings after the conference?	Proportion of SSIs who conducted at least one Parent/Caregiver Training	13	27%	
	Did certified SSIs hold Safe Sleep Community Baby Showers (CBS)/Crib Clinics (CC)?	Proportion of SSIs who held at least one CBS	15	31%	
		Proportion of SSIs who held at least one CC	15	31%	
	Did certified SSIs meet requirements of training at least 10 professionals and holding 1 CBS or 10 CCs?	Proportion of SSIs who met requirements	11	22%	
	Did certified SSIs fail to provide any post-conference trainings?^3^	Proportion of SSIs who conducted no post-certification trainings	16	33%	
			Pre M (SD)	Post M (SD)	t-test, *p*-value
I	Did knowledge of professionals and/or caregivers change after being trained by a certified SSI?	Change in knowledge on Professional Training Test (10-item) for 338 professionals trained by SSIs	7.2 (1.2)	9.2 (1.1)	t = 20.2, *p* < 0.001
			Pre % Correct	Post % Correct	Mann-Whitney U, *p*-value
		Change in knowledge on Parent/Caregiver Training Test (3-item) for 180 caregivers trained by SSIs			
		Back only to sleep	76%	99%	*p* < 0.001
		Only a safe sleep location	76%	95%	*p* < 0.001
		Only safe items	50%	96%	*p* < 0.001
			Pre % Correct	Post % Correct	McNemar, *p*-value
		Change in knowledge on CBS/CC Test (3-item) for 333 CBS attendees and 52 CC attendees			
		Back only to sleep	81%	95%	*p* < 0.001
		Only a safe sleep location	88%	97%	*p* < 0.001
		Only safe items	73%	92%	*p* < 0.001
			*N*	%	
M	Do SSIs maintain certification?	Number of SSIs who attend recertification training 1 year after certification	45	92%	

Data is reported for SSIs trained in FY 2022 (Fall 2021: October 1, 2021–September 30, 2022; Spring 2022: June 1, 2022–May 31, 2023). R, Reach; E, Effectiveness; A, Adoption; I, Implementation; M, Maintenance; SSI, Safe Sleep Instructor; AAP, American Academy of Pediatrics; CBS, Safe Sleep Community Baby Shower; CC, Safe Sleep Crib Clinic. ^1^Other: examples include, student, surveyor, director, human resources, and administer. ^2^An SSI may have conducted any combination of these trainings. ^3^Post-conference trainings refer professional training, caregiver training, CBS or CC.

#### 3.1.2 RE-AIM: effectiveness

Forty-four SSI trainees passed the SSI Training post-test with a score of 90% or greater. Five trainees (4 Fall; 1 Spring) scored less than 90% and received remediation. All participants passed the crib demonstration with a score of 90% or greater. All 49 trainees certified as SSIs.

#### 3.1.3 Kirkpatrick: reaction

All 49 (100%) SSI trainees completed the post-conference evaluation. Overall, trainees agreed the topics were relevant (M = 4.98; SD = 0.14) and the training was worthwhile (M = 4.96; SD = 0.20). Trainees were very satisfied with the training (M = 4.94; SD = 0.24).

#### 3.1.4 Kirkpatrick: learning

On the evaluation, SSI trainees reported the materials presented were understandable (M = 4.96; SD = 0.20), most information was new (M = 3.92; SD = 1.05) and they gained insight into safe infant sleep (M = 4.88; SD = 0.33). Trainees were extremely likely to intend to provide safe sleep education in their community (M = 4.92; SD = 0.27). When asked about post-conference training requirements, trainees felt prepared to facilitate a Parent or Caregiver Training (M = 4.78; SD = 0.46), a Professional Training (M = 4.63; SD = 0.60), a Safe Sleep Crib Clinic (M = 4.73; SD = 0.53), and a Safe Sleep Community Baby Shower (M = 4.41; SD = 0.81).

### 3.2 Post-conference

#### 3.2.1 RE-AIM: adoption

Since certification, 24 (49%) SSIs conducted professional trainings, of those, 16 (67%) trained at least 10 professionals. Thirteen (27%) SSIs conducted parent/caregiver trainings and of these, 6 (46%) trained at least 10 parent/caregivers. Fifteen (31%) SSIs hosted at least one CBS. Fifteen (31%) SSIs facilitated CCs, with 4 (27%) facilitating at least 10. Eleven (22%) SSIs completed the post-conference trainings at levels requested.

#### 3.2.2 Kirkpatrick: behavior

Of the 49 SSIs certified, 16 (33%) conducted no post-certification trainings.

#### 3.2.3 RE-AIM: implementation

After being trained by a certified SSI, statistically significant increases in participant knowledge were observed between Professional Training (*n* = 338), Parent/Caregiver Training (*n* = 180), and CBS/CC (*n* = 385) pre- and post-tests.

#### 3.2.4 RE-AIM: maintenance

Forty-five (92%) SSIs recertified for Fiscal Year 2023. Of the 33 SSIs who provided at least one training, 31 (94%) recertified. Of the 16 who conducted no post-certification trainings, 14 (89%) recertified with commitment to provide trainings at requested levels in Fiscal Year 2023.

## 4 Discussion

Despite promotion of evidence-based risk reduction strategies for nearly 30 years, SUID remains a primary cause of infant mortality. Many sleep-related deaths are likely due to modifiable factors. The Kansas State Child Death Review Board reported 98% of SUID deaths in 2020 had external contributing factors such as unsafe sleep location (35). While safe sleep interventions have shown some success (17), implementation science is needed to facilitate the translation of the AAP Safe Sleep Recommendations into behavioral practice (25).

This study offers a preliminary application of dissemination and implementation science to the promotion of the AAP Safe Sleep Recommendations through a train-the-trainer program. In terms of RE-AIM (29, 30), findings regarding SSI program success were mixed. Reach (SSIs trained), Effectiveness (change in knowledge of SSIs), Implementation (change in knowledge of those trained by SSIs) and Maintenance (recertification of SSIs) were high, suggesting merits in terms of ability to build a program infrastructure for dissemination of safe sleep education. However, Adoption (post-certification trainings provided by SSIs) fell far below expectations with 22% of SSIs providing training at the required levels. Based on Kirkpatrick Behavior (31), 33% of certified SSIs engaged in no post-certification activity suggesting no individual or organizational impact from the SSI training for those participants.

Low Adoption, when training effectiveness has been established, is an indicator that program modifications are needed to ensure uptake. Variables impacting Adoption are likely multifaceted and involve a confluence of program, individual, organizational and environmental factors. Most SSIs were able to engage in some level of post-certification training, but few achieved the number requested. In terms of individual factors, the variables assessed related to Kirkpatrick Reaction and Learning (31), such as perceived relevance and confidence, were high for all trainees. Differences in SSIs’ professions may have acted as a facilitator or barrier in training certain audiences or engaging in different training formats. Other demographic or individual characteristics not measured may have also impacted Adoption but were beyond the scope of this study.

In terms of environmental factors, population density may have limited access to both professionals and perinatal persons in frontier areas of Kansas. Further, during FY22, some COVID-19 restrictions were still in effect and may have limited ability to conduct trainings and community events, especially for pregnant participants (32, 36–38). It is also possible that other community-level factors, such as cultural beliefs about infant sleep, impacted SSIs’ ability to engage the local populace.

Many SSIs engaged in certification due to their job (39), therefore, organizational factors must also be taken into consideration regarding Adoption. For example, many healthcare and public health organizations experienced reductions in maternal/infant services due to shifting of staff responsibilities to focus on COVID-related activities (e.g., vaccination, enhance safety measures) (36). While priority was given to SSI applicants who expressed organizational support, the level of institutional buy-in for safe sleep initiatives was not assessed. Competing or conflicting priorities at the organizational level may have also impacted an SSI’s ability to conduct trainings. Strategies to improve Adoption should address perceived value of providing safe sleep training from organization/employer’s perspective. Examination of additional characteristics, including organization size, type and structure may further elucidate barriers to engaging in post-conference dissemination.

To increase Adoption, SSIs must have the capacity, resources and expertise necessary to comply with program requirements. These factors are necessary to ensure program fidelity, so all SSIs deliver consistent messages regarding safe infant sleep practices. Evaluation of Adoption in subsequent years can help determine whether challenges were pandemic-related or reflect additional barriers in disseminating safe sleep education. Qualitative interviews with current SSIs may also help identify barriers and facilitators to Adoption. Mixed methods designs are particularly useful for initiatives where the goal is to learn about adaptability and use (40), which can support informed decision-making about implementation and translation (41). In addition to interviews, observation of SSIs conducting training (e.g., CBS) may identify program structure or contextual factors that could help improve post-certification dissemination activities. A better understanding of impediments to Adoption may assist with identifying pragmatic ways to tailor the program in different settings.

Once SSI needs are better understood, program modifications should be considered in determining the best way to increase Adoption. For example, within the SSI program, there may be benefits to offering “tracts” where SSIs specialize in educating either professionals or families as many SSIs had success with one group but not the other. Allowing for adaptability of the program, to better meet the needs of SSIs and their organizations is necessary to enhance program effectiveness. Throughout the certification training, SSIs are challenged to consider adaptations to best meet their community needs, such as addressing local cultural beliefs and identifying key partners to reach priority populations. However, the lower levels of Adoptions suggest broader adaptations of the SSI program may be needed, especially as the program is integrated into new settings.

It may also be useful to partner with external organizations whose staff are seeking SSI certification to establish policies and infrastructure to ensure institutional support for success. For example, hospitals that have or are working on the Cribs for Kids’ National Safe Sleep Hospital Certification (42) may benefit from having an SSI on staff. The Hospital Certification requires a system-wide policy, annual staff training, education to families and provision of cribs to families in need, all of which could be supported by a certified SSI. Better understanding partnership opportunities can facilitate program changes most likely to increase Adoption by SSIs.

With over 35,000 births in Kansas annually, it will be necessary to reach a critical mass to eliminate sleep-related infant deaths. Utilizing implementation science strategies to enhance Adoption is necessary to strengthen sustainability of the program as cost of retaining SSIs is much lower than identifying and certifying new trainees. In addition to strategies outlined above, behavioral measures regarding professional dissemination of safe sleep information following training by an SSI, and use of safe sleep practices following Safe Sleep CBS/CCs could be incorporated into future studies. Iterative applications of the RE-AIM framework and Kirkpatrick Evaluation Model should be applied to determine impact of strategies to increase Adoption over the course of program implementation (29–31).

### 4.1 Limitations

This study has several limitations. The SSI conference took place in, and most participants were from, a Midwestern state in the US. This may impact generalizability of findings related to conferences in other locations. Evaluation of Reach was limited as the distribution of SSI certification training invitations included listservs and professional groups that could not be quantified, and recipients were encouraged to share the invitation with others. Many of the variables assessed were collected based on self-report (e.g., self-assessed knowledge change, behavioral intentions) and may have been impacted by social desirability bias. There was no follow-up to assess long-term knowledge change (professionals, parents/caregivers) or behavior change (perinatal persons) following training by the SSIs. Kirkpatrick Results (31) were unable to be assessed with program data, however future reports on state Pregnancy Risk Assessment Monitoring System (PRAMS) findings related to infant sleep practices may offer some insight. In addition, the ultimate outcome of this intervention, reduced SUID rates, was beyond the scope of this evaluation due to the delay in availability of infant mortality data. Long-term SUID trends will be monitored, using state vital statistics reports, though causation cannot be assumed.

## 5 Conclusion

This study offers a preliminary application of dissemination and implementation science to the promotion of the AAP Safe Sleep Recommendations through a train-the-trainer program. Utilizing the RE-AIM framework and Kirkpatrick Evaluation Model allowed investigators to apply well-established models to clearly identify strengths and limitations of the current program (29–31). The SSI certification was successful in terms of Reach, Effectiveness, Implementation and Maintenance. Kirkpatrick Reaction and Learning also had positive findings. Adoption was less than expected. Enhancing adaptability of the certification requirements may increase success and sustainability of the program. Further refinement of program expectations enhanced post-conference support for Adoption activities, and opportunities for tailoring may enhance Adoption efforts. Future studies will further assess characteristics that impact Adoption in order to enhance promotion of SUID risk reduction strategies.

## Data Availability

The raw data supporting the conclusions of this article will be made available by the authors, without undue reservation.
